# Impact of Nutritional Status on Gastroenteropancreatic Neuroendocrine Tumors (GEP-NET) Aggressiveness

**DOI:** 10.3390/nu10121854

**Published:** 2018-12-01

**Authors:** Luigi Barrea, Barbara Altieri, Giovanna Muscogiuri, Daniela Laudisio, Giuseppe Annunziata, Annamaria Colao, Antongiulio Faggiano, Silvia Savastano

**Affiliations:** 1 Dipartimento di Medicina Clinica e Chirurgia, Unit of Endocrinology, Federico II University Medical School of Naples, Via Sergio Pansini, 5, 80131 Naples, Italy; altieri.barbara@gmail.com (B.A.); giovanna.muscogiuri@gmail.com (G.M.); daniela.laudisio@libero.it (D.L.); colao@unina.it (A.C.); afaggian@unina.it (A.F.); sisavast@unina.it (S.S.); 2 Division of Endocrinology and Diabetes, Department of Internal Medicine I, University Hospital, University of Wuerzburg, 97080 Wuerzburg, Germany; 3 Department of Pharmacy, University of Naples “Federico II”, Italy; Via Domenico Montesano, 49, 80131 Naples, Italy; giuseppe.annunziata@unina.it

**Keywords:** gastroenteropancreatic neuroendocrine tumors, nutrition, tumor aggressiveness, Mediterranean diet, bioelectrical impedance analysis

## Abstract

Neuroendocrine tumors (NETs) are rare neoplasms mostly originating from the gastroenteropancreatic tract (GEP-NETs). Data regarding nutritional status in GEP-NET patients are limited. The aim of the study was to investigate the nutritional status and adherence to the Mediterranean Diet (MD) in GEP-NET patients and to correlate them with tumor aggressiveness. A cross-sectional case-control observational study was conducted enrolling 83 patients with well-differentiated G1/G2 GEP-NETs after resection, as well as 83 healthy subjects, age, sex and body mass index-matched. Nutritional status was assessed by evaluating with Bioelectrical Impedance analysis and its phase angle (PhA), adherence to the MD according to PREDIMED score, dietary assessment, anthropometric parameters, and clinico-pathological characteristics. GEP-NET patients consumed less frequently vegetables, fruits, wine, fish/seafood, nuts, and more frequently red/processed meats, butter, cream, margarine, and soda drinks than controls. Patients with more aggressive disease presented a lower adherence to MD according to PREDIMED categories in comparison to G1, localized and free/stable disease status. A smaller PhA value and a lower PREDIMED score were significantly correlated with G2 tumor, metastases, and progressive disease. To the best of our knowledge, this is the first study reporting an association between nutritional status and tumor aggressiveness in a selected group of GEP-NETs. Moreover, higher intakes of food of MD, may represent a potential tool for prevention of tumor aggressiveness. Thus, a skilled nutritionist should be an integral part of the multidisciplinary management of GEP-NET patients.

## 1. Introduction

Neuroendocrine tumors (NETs) are rare neoplasms, with an estimated annual incidence of ~6.9 *per* 100,000. More than 50% of these tumors originate from the digestive system and are referred to as gastroenteropancreatic NETs (GEP-NETs) [1]. The majority of GEP-NETs are diagnosed as incidental findings or due to clinical manifestation related to tumor mass (non-functioning NETs) [2]. In about 30% of cases, symptoms are related to hypersecretion of hormones and other biologically active molecules (functioning NETs) which can induce different metabolic and gastrointestinal impairments, such as hypoglycemia, hyperglycemia, peptic ulcers, diarrhea, steatorrhea, and altered gastrointestinal motility [3]. Thus, tumor hypersecretion as well as the surgical approach in altering the anatomy of the gastrointestinal tract, the somatostatin analogs treatment affecting the gut’s secretion, motility and absorption, and systemic chemotherapy causing anorexia and weight loss, may all lead to an alteration of the nutritional status in patients affected by GEP-NET [4].

Despite the nutritional status being largely affected in GEP-NET patients, only few studies have investigated this topic. Recently, it was demonstrated that metabolic profile in patients with non-functioning GEP-NET was associated with higher ki67% index and larger tumor size [5]. In contrast to this, a large analysis using the Nationwide Inpatient Sample showed that obesity could represent a protective factor against inpatient mortality in abdominal NET patients [6]. The same study also reported that malnutrition correlated with nearly 5-fold higher odds of inpatient mortality in this group of patients [6]. It has been suggested that a poor nutritional status may negatively impact the clinical outcome of patients with NET, extending the duration of hospitalization and reducing the overall-survival [7,8]. Moreover, malnourished NET patients showed a worse response to transcatheter arterial chemoembolization of liver metastases [9]. Maasberg et al. observed an association between nutritional status and body composition evaluated through Bioelectrical Impedance Analysis (BIA) and its derived parameter Phase Angle (PhA) in NET patients [8], as already reported in other cancer types [10,11]. Particularly, the authors reported that malnutrition significantly correlated with poorer BIA parameters and decreased PhA value [8]. No further details on nutritional status, dietary pattern, and body composition in GEP-NET patients have been reported.

Evidence suggests that nutritional factors, such as the scarce adherence to the Mediterranean diet (MD), could influence the aggressiveness of different tumor types, such as prostate, bladder, and breast cancer [12,13,14], in addition to representing a dietary pattern suitable in both prevention and mortality reduction of several cancer types [15]. Moreover, it has been reported that a low adherence to the MD correlated with an increased risk of metabolic syndrome [16], that, as above mentioned, was associated with larger and higher proliferative tumor in GEP-NET patients [5]. Recently, an association between PhA measurements and adherence to the MD independently from confounding factors, such as sex, age, and body weight in healthy subjects was observed [17]. Nowadays, nutritional guidelines in GEP-NET patients are still missing and there is no evidence regarding the association between the adherence to the MD and its potential association with tumor aggressiveness in patients with GEP-NET. Thus, the evaluation of PhA value and the adherence to the MD could be particularly useful to predict the nutritional status of GEP-NET patients.

The aim of this case-control, cross-sectional study was to investigate the nutritional status, including adherence to the MD and dietary pattern, in a selected group of GEP-NET patients and to evaluate the association of the nutritional status with different markers of tumor aggressiveness.

## 2. Materials and Methods

### 2.1. Design and Setting

This paper presnents a cross-sectional case-control observational study carried out at the European Neuroendocrine Tumor Society (ENETS) Center of Excellence Multidisciplinary Group for Neuroendocrine Tumors, Department of Clinical Medicine and Surgery, Unit of Endocrinology, University “Federico II” of Naples. Both patients and controls were enrolled from January 2017 to July 2018. The work was conducted in accordance with the Code of Ethics of the World Medical Association (Declaration of Helsinki) for experiments involving humans, and approved by the Ethical Committee of the University of Naples “Federico II” Medical School (n. 201/17). The protocol was explained to both patients and controls, and a written informed consent was obtained. This cross-sectional observational study was registered at clinicaltrials.gov with the number NCT03592940.

### 2.2. Population Study

The study was conducted on 83 adult patients affected by GEP-NET out of 172 unselected Caucasian patients with a diagnosis of NET attending the ENETS Centers of Excellence Multidisciplinary Group for Neuroendocrine Tumors, University “Federico II”, Naples. To improve the power of the study and the homogeneity of the investigated cohort, only patients with the following criteria were included:

- Histological diagnosis of well-differentiated, low grade (G)1 and G2 GEP-NET, including sporadic tumors or patients with multiple endocrine neoplasia type 1 (MEN1) syndrome, according to classification of by the World Health Organization (WHO) [18];
- Non-functioning GEP-NET patients that were treatment-naïve (evaluated at the moment of the diagnosis or before starting any medical treatment), or that discontinued Somatostatin Analogues (SSAs) for more than 6 months or after endoscopic surgery performed more than 6 months before the visit;
- Functioning GEP-NET patients who underwent endoscopic tumor resection and who were biochemically free of disease for more than 6 months and who had not resumed medical treatment;

Patients with one or more of the following criteria were excluded from the study:

- Histological diagnosis of well-differentiated/high grade G3 GEP-NET or poorly-differentiated neuroendocrine carcinomas (NEC) according to WHO classification [18], since it has been shown that patients with G3 tumors were at risk of malnutrition [8];
- Diagnosis of bronchial or thymic NET, medullary thyroid cancer, Merkel cell carcinoma, pheochromocytoma/paraganglioma;
- Ongoing medical treatment, including SSAs or targeted therapy, at the moment of the visit, since they could affect the gastrointestinal secretory, motor, and absorptive functions or cause anorexia and liver toxicity [5];
- Patients who had undergone major surgery, since it could change the anatomy of the gastrointestinal tract;
- Patients with functioning GEP-NET that had been treated with curative surgery for less than 6 months before the visit;
- Patients with functioning GEP-NET that had not been treated at the moment of the visit, since the secretion of hormones, peptides and amines could cause malabsorption, diarrhea, steatorrhea and altered motility of the gastrointestinal tract [5];
- Patients on a hypocaloric diet in the last three months or specific nutritional regimens, including vegan or vegetarian diets and vitamin/mineral or antioxidant supplementation;
- Presence of clinical conditions that could influence fluid balance and metabolism, including diabetes mellitus, hypertension, liver or renal failure, acute or chronic inflammatory diseases, history of cancer, based on a complete medical examination and laboratory investigations;
- Current administration of medicaments that could influence the fluid balance, including non-steroidal anti-inflammatory drugs, hormone replacement therapy, diuretics or laxative;
- Abuse of alcohol defined by the Diagnostic and Statistical Manual of Mental Disorders (DSM)-V criteria [19];

Patients with pacemakers due to the potential interference with the device.

**Figure 1:**
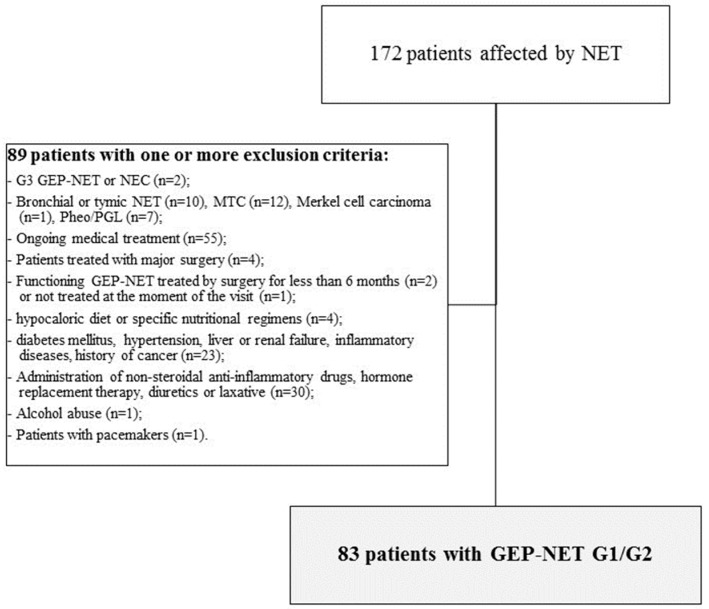
Flow chart of the studied subjects. A total of 83 patients affected by well differentiated GEP-NET G1/G2 selected from 172 patients with NET attending the ENETS Centers of Excellence Multidisciplinary Group for Neuroendocrine Tumors, University “Federico II”, Naples. Abbreviation: NET, Neuroendocrine Tumor; GEP-NET, Gastroenteropancreatic NET; MTC, medullary thyroid cancer; Pheo/PPG, Pheochromocytoma/paraganglioma; ENETS, European Neuroendocrine Tumor Society.

Eighty-three patients were enrolled together with healthy volunteers from the hospital and employees from the same geographical area. Controls were matched by age, sex, and Body Mass Index (BMI) and none had a history of cancer, diabetes mellitus, hypertension, liver or renal failure, inflammatory disease, alcohol abuse and none of them consumed medicaments. To avoid the overlapping enrollment, none of the controls contemporarily participated in other trials during the period of this study. All the measurements were performed between 8 and 12 AM. All subjects were measured after an overnight fast. The flow chart of the studied subjects is shown in Figure 1.

### 2.3. Clinicopathological Characteristics of the Tumor

Clinicopathological characteristics, such as primary tumor site and size, tumor stage, mitotic rate, ki67 index, metastases, familiar history of MEN1, hormonal secretion, comorbidity, treatment and follow-up, were collected for all patients.

Tumor size (mm) was defined as the maximum tumor diameter in the pathological specimen or in the last computed tomography (CT) scan/magnetic resonance imaging (MRI) when the patient had not undergone surgery or when the patient had multiple pancreatic nodules in case of MEN1. For these patients, the diameter of the biggest pancreatic lesion was considered for the tumor size. Only in a few cases (*n* = 3) was the tumor size not defined since the primary lesion had not been found. Tumor stage at diagnosis was classified according to the ENETS criteria [20]. According to this, patients were classified as those with localized disease (stage I–III) and patients with advanced disease (presence of metastases, stage IV).

For the diagnosis of NET, immunohistochemistry for chromogranin A, synaptophysin and ki67 were performed for all formalin-fixed paraffin-embedded tissue samples deriving from biopsy or surgery of the primary tumor and/or metastases [21]. Particularly, the evaluation of the mitotic rate and ki67% index were performed as previously reported [22] according to ENETS criteria [20]. Additionally, according to WHO classification [18], all GEP-NETs were divided into well-differentiated or poorly differentiated malignant neoplasms named neuroendocrine carcinomas (NECs). In this study, only patients with well-differentiated/low grade GEP-NETs, graded as G1 (ki67% ≤2% and mitoses <2) or G2 (ki67% 3–20% and mitoses 2–20) were included [18].

At the time of the visit, disease status was defined as “disease free”, when there was no biochemical and morphological evidence of the disease after tumor resection, “stable disease” or “progressive disease” according to RECIST 1.1 criteria [23].

### 2.4. Lifestyle Habits

Lifestyle habits, including physical activity level and smoking habits, were investigated by a standard questionnaire. Physical activity levels were expressed according to whether the participant habitually engaged at least 30 min/day of aerobic exercise (YES/NO). Subjects were considered as “current smokers” when they smoked at least one cigarette per day, “former smokers” when having stopped smoking at least one year before the interview, and “non-current smokers”. Former and non-current smoker were considered as “no-smoker” for the analyses.

### 2.5. Dietary Assessment

As widely reported previously [24,25,26,27], data were obtained during a face-to-face interview between the patient and a qualified nutritionist. In detail, the dietary interview enabled the quantification of food and drinks by using a photographic food atlas (≈1000 photographs) of known portion sizes to ensure accurate completion of the records [28]. Dietary data, including beverage intakes and alcohol consumption, were collected by 7-day food records that were evaluated by a nutritionist. Data were stored and processed later, using a specific software (Terapia Alimentare Dietosystem^®^ DS-Medica, (http://www.dsmedica.info) that calculate daily caloric intake and the quantities of macronutrients (animal and plant protein; total, complex and simple carbohydrates; total fat, saturated fatty acid (SFA), monounsaturated fatty acids (MUFA), polyunsaturated fatty acids (PUFA): n-6 PUFA, n-3 PUFA).

### 2.6. Adherence to the Mediterranean Diet

As previously reported [17,24,25,26,29,30,31,32], the adherence to the MD was evaluated using the 14-item questionnaire for the assessment of PREvención con DIeta MEDiterránea (PREDIMED) [33]. A qualified nutritionist administered the questionnaire during a face-to-face interview to all the enrolled subjects. Briefly, for each item scores 1 and 0 were assigned; the PREDIMED score was calculated as follows: 0–5, lowest adherence; score 6–9, average adherence; score ≥10, highest adherence [33].

### 2.7. Anthropometric Measurements and Blood Pressure

Anthropometric measurements were obtained with subjects wearing light clothes and without shoes. BMI was calculated by weight and height (weight (kg) divided by height squared (m^2^), kg/m^2^). Height was measured to the nearest 1 cm using a wall-mounted stadiometer (Seca 711; Seca, Hamburg, Germany). Body weight was derived to the nearest 50 g using a calibrated balance beam scale (Seca 711; Seca, Hamburg, Germany). Subjects were classified by BMI according to WHO’s criteria as normal weight (BMI 18.5–24.9 kg/m^2^), overweight (BMI 25.0–29.9 kg/m^2^), grade I obesity (BMI 30.0–34.9 kg/m^2^), grade II obesity (BMI 35.0–39.9 kg/m^2^), grade III obesity (BMI ≥ 40.0 kg/m^2^) [34]. Waist Circumference (WC) was measured to the closest 0.1 cm at the natural indentation or at a midway level between lower edge of the rib cage and iliac crest if no natural indentation was visible using a non-stretchable measuring tape, in line with the National Center for Health Statistics (NCHS) [35]. In all individuals systolic (SBP) and diastolic (DBP) blood pressure were measured three times, every two min after the subject had been sitting for at least 10 min, with a random sphygmomanometer (Gelman Hawksley Ltd., Sussex, UK). The mean of the second and third reading was recorded.

### 2.8. Assay Methods

Samples were collected in the morning between 8 and 10 a.m., after an overnight fast of at least 8 h and stored at −80 °C until being processed. All biochemical analyses including fasting plasma glucose, total cholesterol, fasting plasma triglycerides were performed with a Roche Modular Analytics System in the Central Biochemistry Laboratory of our Institution. Low-density lipoprotein (LDL) cholesterol and high-density lipoprotein (HDL) cholesterol were determined by a direct method (homogeneous enzymatic assay for the direct quantitative determination of LDL and HDL cholesterol).

### 2.9. Bioelectrical Impedance Analysis

Bioelectrical impedance analysis was performed using a BIA phase-sensitive system by experienced observers (an 800-µA current at a frequency single-frequency of 50 kHz BIA 101 RJL, Akern Bioresearch, Florence—Italy) [36], as already amply reported in previous studies [17,29,37]. Based on the European Society of Parenteral and Enteral Nutrition (ESPEN) guidelines [38], all participants were supine with limbs slightly spread apart from the body, had refrained from eating, drinking, and exercising for six hours with no alcohol within 24 h before testing. Shoes and socks were removed and contact areas were scrubbed with alcohol immediately before electrode placement. Electrodes (BIATRODES Akern Srl; Florence—Italy) were placed on the dorsal surface of the right hand proximal to the phalangeal–metacarpal joint and on the superior surface of the right foot distal to the transverse arch. Sensor electrodes were placed on the right wrist at the midpoint between the distal prominence of the radius and ulna, and on the right ankle between the medial and lateral malleoli, as described by Kushner et al. [39]. All measurements were performed under strictly standardized conditions by the author, using the same device in order to avoid interobserver and interdevice variability. The instrument was routinely checked with resistors and capacitors of known values. Reliability for within-day and between-day measurements by the same observer were <2.7% for resistance (R), <2.9% for reactance (Xc), and <3.1% for R, <2.4% for Xc, respectively. The coefficient of variation (CV) of repeated measurements of R and Xc at 50 kHz was assessed in 14 patients (7 males and 7 females) by the same observer: CVs were 1.9% for R and 1.8% for Xc. The PhA was derived from conditions under 50 kHz according to the following formula: PhA (°, degrees) = arctangent Xc/R ((Xc/R) × (180/*π*)).

### 2.10. Statistical Analysis

The data distribution was evaluated by the Kolmogorov–Smirnov test and the abnormal data were normalized by logarithm. All variables were logarithmically transformed and back-transformed for presentation in tables and figures. The chi-square (χ^2^) test was used to determine the significance of differences in the frequency distribution. Differences between GEP-NETs patients and the control group were analyzed by Student’s paired *t*-test, while the differences among the several parameters with the disease status were analyzed by Student’s unpaired *t*-test, followed by Bonferroni post hoc analysis. *p* values < *0.05* were considered as statistically significant.

Proportional Odds Ratio (OR) models, *p*-value, 95% Interval Confidence (IC), and R^2^, were performed to assess the association among quantitative variables (grading system and metastasis). Multinomial logistic regression, χ^2^, *p*-value, and Akaike Information Criterion (AIC), were performed to model the relationship between the several parameters with the three groups of disease status (disease free, stable disease, and progressive disease). The correlations between the different variables were performed using Pearson *r* correlation coefficients. Receiver operator characteristic (ROC) curve analysis was performed to determine sensitivity and specificity, area under the curve (AUC), and IC, as well as cut-off values for PhA and PREDIMED score in detecting Sartorius HS score above the median values in the HS patients. Test AUC for ROC analysis was also performed. For α level the 0.05 type I error was selected and for β level the 0.20 type II error was selected. In these last analyses, only variables with a *p*-value < 0.05 in the univariate analysis (partial correlation) were entered. Variables with a variance inflation factor (VIF) >10 were excluded to avoid multicollinearity. Values ≤ 5% were considered statistically significant. Data were analyzed using the MedCalc^®^ package (Version 12.3.0 1993–2012 MedCalc Software bvba-MedCalc Software, Mariakerke, Belgium) and SPSS Software (PASW Version 21.0, SPSS Inc., Chicago, IL, USA).

## 3. Results

### 3.1. Patient Population

A total of 83 patients (F:M = 43:40), mean age 56.66 (18–80) years, affected by GEP-NET were included in the study. Mean size of the tumor was 24.96 ± 23.79 mm. Primary NET were located in the pancreas (*n* = 47, 56.6%), stomach (*n* = 12, 14.5%), small intestine (*n* = 14, 16.9%), appendix (*n* = 5, 6%), colon (*n =* 2, 2.4%) and in a few cases the primary site was unknown (*n* = 3, 3.6%). The majority of patients had non-functioning GEP-NET (*n* = 75, 90.4%). Among patients with pancreatic NET, 39 were non-functioning (83%), 5 were insulinomas (10.6%), 2 were gastrinomas (4.2%) and 1 was VIPoma (2.2%). All patients with functioning pancreatic NET underwent endoscopic surgery of the lesion at least 6 months before the visit. Twenty two patients (26.5%) had a MEN1 syndrome.

According to the pathological parameters, the mitotic rate and ki67% index, all GEP-NET were classified as well-differentiated tumor G1 (*n* = 48, 57.8%) or G2 (*n* = 35, 42.2%). At diagnosis, 22 patients (26.5%) had metastases (stage IV), the majority of them in the liver. At the moment of the visit considered for the study, 34 patients (41%) were disease free, 28 (33.7%) had stable disease and 21 (25.3%) had progressive disease according to the RECIST1.1 criteria.

### 3.2. Nutritional Status in GEP-NET Patients and Control Group

Clinical and anthropometric characteristics, lifestyle habits, blood pressure, metabolic profile, and bioelectrical parameters deriving from BIA of GEP-NET patients compared to controls are shown in Table 1. To note, GEP-NET patients smoked less (*p* = 0.005), presented higher blood pressure values and a worse metabolic profile (*p* < 0.001), and had smaller PhA according to gender (*p* < 0.001) in comparison to the control group (Table 1).

Data on Mediterranean food frequencies were analyzed by using the 7-day food records. Even though no differences in energy intake were observed between the two groups, GEP-NET patients consumed a lower quantity of plant protein (*p =* 0.003), complex carbohydrate (*p* < 0.001), MUFA (*p =* 0.009) and n-3 PUFA (*p* < 0.001), and higher quantity of simple carbohydrate (*p* < 0.001) and n-6 PUFA (*p* < 0.001) than control subjects (Table S1).

Analyzing in details the frequency of the assumed dietary components included in the PREDIMED questionnaire, GEP-NET patients consumed vegetables less frequently (*p =* 0.005), fruits (*p =* 0.001), wine (*p =* 0.013), fish/seafood (*p =* 0.005), nuts (*p <* 0.001), and more frequently red/processed meats (*p =* 0.017), butter, cream, margarine (*p =* 0.001) and soda drinks (*p =* 0.003); Table 2. Therefore, the PREDIMED score was significantly lower in GEP-NET patients compared to control group (6.18 ± 2.25 vs. 7.39 ± 2.54; *p =* 0.001). The PREDIMED score was positively correlated with PhA both in males and in females, in both GEP-NET patients (r = 0.724, *p* < 0.001 and r = 0.682, *p* < 0.001; respectively) and control (r = 0.919, *p* < 0.001 and r = 0.859, *p* < 0.001; respectively).

Regarding the adherence to the MD, only 4.8% of GEP-NET patients vs 26.5% of controls presented a high adherence to this dietary regimen (*p =* 0.001); Table 2.

### 3.3. Nutritional Status in GEP-NET Patients According to Tumor Grading, Stage and Disease Status

Differences in age, anthropometric measurement, blood pressure, metabolic profile, bioelectrical variables, and nutritional assessment in the GEP-NET patients grouped by grading G1/G2 and stage are summarized in Table 3. Interestingly, patients with GEP-NET G2 and stage IV had significantly higher levels of SBP, fasting glucose, total and LDL cholesterol, triglycerides, and lower levels of HDL cholesterol as well as a lower PREDIMED score and PhA in comparison to patients with localized GEP-NET G1 (Table 3).

Similar results were observed also when these parameters were correlated with disease status. GEP-NET patients with progressive disease showed a significantly worse metabolic profile, a smaller PhA (*p* < 0.001), and a significantly lower PREDIMED score (*p* < 0.001) in comparison to patients who were free of the disease or with stable disease (Table 4).

**Figure 2:**
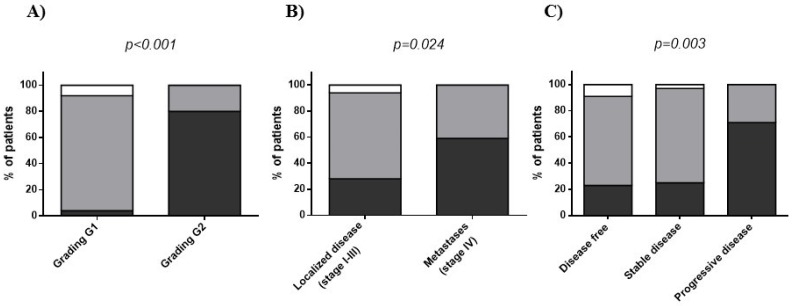
Adherence to the Mediterranean Diet according to PREDIMED categories in GEP-NET patients classified by tumor grade G1/G2 (**A**), stage (**B**), and disease status (**C**). Abbreviation: GEP-NET, Gastroenteropancreatic Neuroendocrine Tumor; PREDIMED, PREvención con DIeta MEDiterránea; MD, Mediterranean Diet.

When classified GEP-NET patients based on tumor grade G1/G2, stage and disease status, the majority of patients with aggressive disease (GEP-NET G2, stage IV and progressive disease) presented a low adherence to the MD according to PREDIMED categories (Figure 2 and Table S2).

In details, 28 out 35 (80%) patients with G2 GEP-NET, 13 out 22 (59%) patients with metastases and 15 out 21 (71.4%) patients with progressive disease had significantly lower adherence to the MD (Figure 2). On the contrary, 87.5%, 65.5%, and 67.7% of patients with GEP-NET G1, localized disease and free of disease, respectively, had an average adherence to the MD. Not one of the patients with aggressive tumor had a high adherence to the MD according to PREDIMED categories (Figure 2). No significant differences were observed when these patients were classified for gender, smoking and physical activity (Table S2).

### 3.4. Correlation between Tumor Aggressiveness and Nutritional Status in GEP-NET Patients

Different markers, such as grading, stage, progressive disease, ki67% index, and tumor size, were evaluated to investigate tumor aggressiveness.

To assess the association of grading and stage, a bivariate proportional OR model with age, anthropometric measurement, blood pressure, metabolic profile, bioelectrical variables, and nutritional assessment was performed (Table 5).

Metastatic G2 tumor were significantly associated with higher values of WC (*p =* 0.049 for both), SBP (*p =* 0.018 and *p =* 0.020 for G2 and stage IV, respectively), metabolic profile (*p* < 0.001 for total and LDL cholesterol, *p =* 0.002 and *p =* 0.007 for fasting glucose and triglycerides, respectively, and G2; *p =* 0.004, *p =* 0.003, *p =* 0.005 and *p =* 0.001 for total cholesterol, LDL cholesterol, fasting glucose and triglycerides, respectively, and stage IV), protein consumption (*p =* 0.029 and *p =* 0.042 for G2 and stage IV, respectively), and with lower levels of HDL cholesterol (*p =* 0.044 and *p =* 0.011 for G2 and stage IV, respectively), lower PhA (*p* < 0.001 and *p =* 0.001 for G2 and stage IV, respectively) and PREDIMED score (*p* < 0.001 and *p =* 0.001 for G2 and stage IV, respectively; Table 5). In addition, GEP-NETs G2 were associated with higher consumption of simple carbohydrate (*p =* 0.042) and metastasized disease with lower consumption of plant protein (*p =* 0.002).

A multinomial logistic regression model to assess the association between patients with progressive disease and age, anthropometric measurement, blood pressure, metabolic profile, bioelectrical variables, and nutritional assessment was performed (Table 6). Progressive disease was associated with higher value of WC (*p =* 0.033), blood pressure (*p =* 0.05 and *p =* 0.027 for SBP and DBP, respectively), fasting glucose (*p =* 0.043), triglycerides (*p =* 0.030), and lower HDL cholesterol (*p =* 0.011), PhA (*p =* 0.010), and PREDIMED score (*p =* 0.005).

Correlations among ki67% index and tumor size with age, anthropometric measurement, blood pressure, metabolic profile, bioelectrical variables and nutritional assessment, are summarized in Table S3. ki67% showed significant correlations with all anthropometric measurements (*p =* 0.002 for BMI and *p* < 0.001 for WC), metabolic profile (*p* < 0.001), SBP (*p =* 0.003), PhA and PREDIMED score (*p* < 0.001). Tumor size correlated with SBP (*p =* 0.040), HDL cholesterol (*p =* 0.018), PhA (*p =* 0.001), PREDIMED score (*p* < 0.001), and protein consumption (*p =* 0.036). After adjusting for BMI and WC, all correlations for both ki67% and tumor size were maintained, except those with SBP (Table S3).

Three multiple linear regression analysis models including variables statistically correlated with each oncological category (grading, tumor stage, and disease status) were performed to compare the relative predictive power of the evaluated variables (Table 7).

Model 1 compared the relative predictive power of grading G1/G2 on WC, SBP, fasting glucose, total cholesterol, HDL cholesterol, LDL cholesterol, triglycerides, PhA, PREDIMED score, protein, and simple carbohydrate consumption. Using this model the PREDIMED score entered at the first step (*p* < 0.001), followed by PhA (*p* < 0.001), and simple carbohydrate (*p =* 0.005). Model 2 compared the relative predictive power of metastases (stage IV) on WC, SBP, DBP, fasting glucose, HDL cholesterol, triglycerides, PhA, and PREDIMED score. Using this model, triglycerides entered at the first step (*p* < 0.001), followed by the PREDIMED score (*p* = 0.003). In the model 3, the disease status was better predicted by PREDIMED score (*p* < 0.001) (Table 7).

The multiple linear regression analysis models including variables statistically correlating with ki67% index and tumor size, were reported in Table S4. In the model 1, ki67% index was better predicted by PhA (*p* < 0.001); in model 2 the dimension of lesion was better predicted by PREDIMED score (*p* < 0.001) (Table S4).

**Figure 3:**
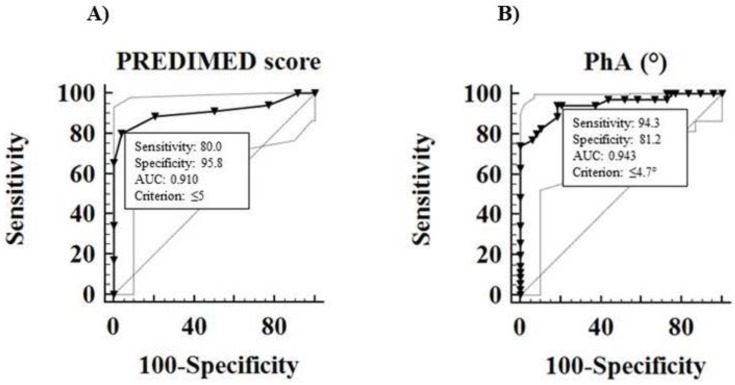
ROC analysis was performed to determine the cut off values of the PREDIMED score (**A**) and PhA (**B**) predictive for the evaluation of increased risk of grading G2 Abbreviation**:** PREDIMED, PREvención con DIeta MEDiterránea; PhA, phase angle; ROC, Receiver operator characteristic.

ROC analysis was performed to determine the cut off values of the PREDIMED score and PhA predictive of high grading, metastases, and disease status. A PREDIMED score ≤ 5 (*p* < 0.001, sensitivity 80%, specificity 95.8%; Figure 3A) and a PhA ≤ 4.7° (*p* < 0.001, sensitivity 94.3%, specificity 81.2%, Figure 3B), could serve as thresholds for significant increased risk of G2 tumor.

**Figure 4:**
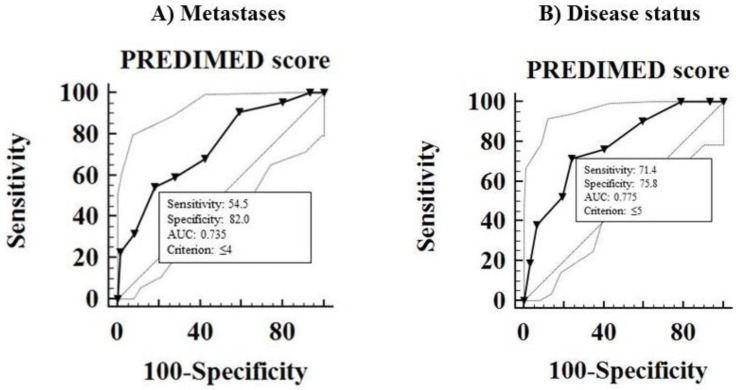
ROC analysis to determine the cut off values of the PREDIMED score for the evaluation of increased risk of metastases (**A**) and progressive disease (**B**). Abbreviation: PREDIMED, PREvención con DIeta MEDiterránea; PhA, phase angle; ROC, Receiver operator characteristic.

A PREDIMED score ≤4 could serve as a threshold for significantly increased risk of metastases (*p* < 0.001, sensitivity 54.5%, specificity 82%; Figure 4A). A PREDIMED score ≤5 was associated with a significant increased risk of progressive disease during follow-up (*p* < 0.001, sensitivity 71.4%, specificity 75.8%; Figure 4B).

## 4. Discussion

GEP-NET patients have been reported to have an impaired nutritional status (the metabolic state, the dietary pattern and body composition), mostly due to excessive secretion of gastrointestinal hormones and peptides, medical treatment, and surgical procedures [4,40]. Different studies have shown that a poor nutritional status negatively impacts the clinical outcome of patients with NET [6,8,9]. Moreover, metabolic profile has been reported to worsen the severity of non-functioning GEP-NETs being associated to a higher ki67% index and larger tumor size [5]. It has been suggested that nutritional factors, such as the adherence to the MD, could influence the aggressiveness of different tumor types, such as prostate, bladder and breast cancer [12,13,14], and that a low adherence to the MD pattern could correlate with an increased risk of metabolic syndrome [16].

In our study we reported the difference of nutritional status, evaluated by BIA and its derived parameter PhA, and dietary pattern between a selected group of GEP-NET patients and healthy control. Moreover, in GEP-NET patients we observed a significant correlation among the nutritional status the adherence to the MD and clinicopathological characteristics, including tumor grade, stage, disease status, ki67% index, and tumor size.

In comparison to healthy controls, patients affected by GEP-NET had a dietary pattern characterized by a significantly lower adherence to the MD, as assessed by PREDIMED score, consuming less frequently vegetables, fruits, wine, fish/seafood, nuts, and more frequently red/processed meats, butter, cream, margarine, and soda drinks. Moreover, in line with the tumor-preventive potential effect of some food containing specific bioactive compounds (e.g., n-3 PUFA, or MUFA) [41], we observed that GEP-NET patients in comparison to healthy controls, had a lower consumption of unsaturated fat that is beneficial for health, were associated with a lower consumption of plant protein and complex carbohydrates and had a preferential consumption of simple carbohydrate and n-6 PUFA. As already reported [16], we observed that GEP-NET patients presented a worse metabolic profile probably as a consequence of a low adherence to the MD. Indeed, the metabolic profile of these patients was characterized by an increase of waist circumference, higher blood pressure values, and significantly higher blood levels of fasting glucose, total and LDL cholesterol and triglycerides, and significantly lower levels of HDL cholesterol, all parameters that are associated with a higher risk of metabolic syndrome. Thus, we confirmed that the worse metabolic parameters correlated with higher ki67% index, as previously reported by our group [5]. However, we did not find significant correlations between these parameters and larger tumor size, except for HDL cholesterol. These contrasting results with our previous study [5] could be due to the fact that in the present study we did not consider the metabolic syndrome per se, but we focused our attention on the nutritional status and dietary pattern.

We observed that GEP-NET patients presented a decreased PhA value in comparison to healthy controls, as already reported in other diseases, such as infection disease, inflammation status, and several cancer types [11]. It has been suggested that the PhA value correlated with disease severity [11] and that a low value could be predictive of impaired prognosis (mortality, disease progression, postoperative complications) in different tumors, such as pancreatic [42], breast [43], lung [44], and colorectal cancer [45]. Moreover, we recently observed that PhA value correlated with the adherence to MD independently of confounding factors [17]. In parallel with these findings, we demonstrated that a decreased PhA, together with a lower adherence to the MD as assessed by a low PREDIMED score, were the major predictors of GEP-NET aggressiveness. The ROC analysis showed that tumor grading G2 was well predicted by a PREDIMED score ≤ 5 and a PhA value ≤ 4.7° with a sensitivity of 80% and 94.3% and a specificity of 95.8% and 81.2%, respectively. Thus, we concluded that a lower adherence to the MD, together with a decreased PhA value and a consequent worse metabolic profile, were associated with an increased GEP-NET aggressiveness, characterized by tumor grading G2, stage IV and progressive disease, also after adjustments for gender, smoking, and physical activity.

However, there are some limits of this study that should be considered. The cross-sectional nature of the study did not allow any causal association to be identified between MD or PhA and GEP-NETs and to clearly determine the prognostic value of the adherence to the MD or of PhA for predicting its clinical severity. Moreover, the suggested cut-off value of the PREDIMED score and PhAs to identify tumor aggressiveness should be viewed with caution until results in larger populations become available to perform an appropriate cross-validation. In addition, expert nutritionists are required for the assessment, execution, and interpretation of BIA measurements, such as PhA. The main strength of this study was the use of the 7-day food records. This method is the “gold standard” in validation studies of different self-administered food frequency questionnaires and allows a more accurate measurement of the dietary and macronutrient intakes compared to other questionnaires [46,47]. In order to improve the power of the study, we increased the homogeneity of the cohort of studied patients by including non-functioning treatment-naïve patients or patients who underwent curative surgery and who were biochemically free of disease for more than 6 months and who had not partaken of medical treatment. Moreover, all patients had a diagnosis of well-differentiated G1/G2 GEP-NET and both GEP-NET patients and matched controls were well characterized.

## 5. Conclusions

In conclusion, to the best of our knowledge, this is the first study to show a novel association between nutritional status and aggressiveness of GEP-NETs in a selected cohort of adult patients. This association potentially extends the benefit of adherence to the MD to GEP-NET patients and suggests that BIA and PhA value may be used as tools for the nutritional management of these patients and as markers of tumor aggressiveness. Moreover, this is the first study to indicate a dietary pattern that may be beneficial for GEP-NET patients and that may modulate the risk of tumor aggressiveness, offering a practical strategy for the management of these patients. Therefore, the assessment of nutritional status should be recommended as good clinical practice in the evaluation of GEP-NET patients, in order to identify high-risk subjects with a more aggressive tumor who could better benefit from a nutritional intervention promoting the Mediterranean food pattern. Thus, a skilled nutritionist should be part of the multidisciplinary health care team in NETs management, adapting the specific nutritional needs to the course of the disease. Future well-designed dietary intervention trials on larger population samples are needed to define specific dietary guidelines for NETs and elucidate the beneficial effects of the MD on the survival outcomes of GEP-NET patients.

## Figures and Tables

**Table 1:** Demographic, clinical, metabolic, and bioelectrical parameters of GEP-NET patients compared to controls.

Parameters	GEP-NET Patients *n*. 83	Control Group *n*. 83	*p*-Value
**Clinical characteristics** Gender-male Age-mean (range)	40 (48.2%) 56 (18–80)	40 (48.2%) 57 (23–82)	0.877 (χ^2^ = 0.02) 0.307
**Lifestyle Habits** Smoking-yes Physical activity-yes	32 (38.6%) 41 (49.4%)	51 (61.4%) 45 (54.2%)	**0.005** (χ^2^ = 7.81) 0.986 (χ^2^ = 0.00)
**Anthropometric measurement** BMI (kg/m^2^) WC (cm)	27.79 ± 5.57 92.76 ± 15.38	27.54 ± 2.84 87.92 ± 10.66	0.859 **0.050**
**Blood pressure** SBP (mmHg) DBP (mmHg)	123.79 ± 12.16 76.99 ± 7.99	118.49 ± 11.93 73.86 ± 6.87	**0.010** **0.007**
**Metabolic profile**			
Fasting Glucose (mg/dL)	109.52 ± 16.51	89.29 ± 11.72	**<0.001**
Total cholesterol (mg/dL)	194.83 ± 41.19	149.08 ± 22.41	**<0.001**
HDL cholesterol (mg/dL)	44.43 ± 14.35	50.45 ± 8.67	**<0.001**
LDL cholesterol (mg/dL)	123.35 ± 40.85	77.20 ± 25.00	**<0.001**
Triglycerides (mg/dL)	135.23 ± 47.81	107.22 ± 26.57	**<0.001**
**Bioelectrical variables**			
R (Ω)	514.23 ± 80.21	523.54 ± 59.99	0.279
Xc (Ω)	42.31 ± 9.50	52.19 ± 8.13	**<0.001**
PhA (°)	4.73 ± 0.88	5.70 ± 0.55	**<0.001**
PhA (°) males	4.69 ± 0.86	5.76 ± 0.60	**<0.001**
PhA (°) females	4.76 ± 0.91	5.65 ± 0.52	**<0.001**

A *p* value in bold type denotes a significant difference (*p* < 0.05). Abbreviation: GEP-NET, Gastroenteropancreatic Neuroendocrine Tumor; BMI, Body Mass Index; WC, Waist Circumference; SBP, Systolic Blood Pressure; DBP, Diastolic Blood Pressure; HDL, High-Density Lipoprotein; LDL, Low-Density Lipoprotein; R, Resistance; Xc, Reactance; PhA, Phase angle.

**Table 2:** Response frequency of dietary components included in the PREDIMED questionnaire of the GEP-NET patients and control group.

Questions of PREDIMED Questionnaire	GEP-NET Patients *n*. 83		Control Group *n*. 83			
	*n*	%	*n*	%	χ^2^	*p*-Values
Use of extra virgin olive oil as main culinary lipid	78	94.0	79	95.2	0.00	1.000
Extra virgin olive oil >4 tablespoons	49	59.0	52	62.7	0.10	0.751
Vegetables ≥2 servings/day	30	36.1	49	59.0	7.83	**0.005**
Fruits ≥3 servings/day	27	32.5	50	60.2	11.72	**0.001**
Red/processed meats <1/day	25	30.1	41	49.4	5.66	**0.017**
Butter, cream, margarine <1/day	63	75.9	40	48.2	12.38	**0.001**
Soda drinks <1/day	34	41.0	54	65.1	8.73	**0.003**
Wine glasses ≥7/week	14	16.9	29	34.9	6.15	**0.013**
Legumes ≥3/week	38	45.8	37	44.6	0.00	1.000
Fish/seafood ≥3/week	18	21.7	36	43.4	7.93	**0.005**
Commercial sweets and confectionery ≤2/week	43	51.8	39	47.0	0.22	0.641
Tree nuts ≥3/week	20	24.1	33	39.8	21.14	**<0.001**
Poultry more than red meats	41	49.4	43	51.8	0.02	0.876
Use of sofrito sauce ≥2/week	33	39.8	31	37.3	0.03	0.873
**PREDIMED categories**						
Low adherence to the MD	30	36.1	28	33.7	0.03	0.870
Average adherence to the MD	49	59.0	33	39.8	5.42	**0.019**
High adherence to the MD	4	4.8	22	26.5	13.18	**0.001**

A *p* value in bold type denotes a significant difference (*p* < 0.05). Abbreviation: GEP-NET, Gastroenteropancreatic Neuroendocrine Tumor; PREDIMED, PREvención con DIeta MEDiterránea; MD, Mediterranean Diet.

**Table 3:** Demographic, clinical, metabolic, and bioelectrical parameters in the GEP-NET patients according to tumor grading and stage.

Parameters	G1 *n*. 48	G2 *n.* 35	*p*-Value	Stage I-III (Localized Disease) *n*. 61	Stage IV (Metastases) *n*. 22	*p*-Value
**Age (years)**	54.85 ± 17.31	59.14 ± 12.64	0.103	55.72 ± 16.82	59.27 ± 11.37	0.123
**Anthropometric measurement**						
BMI (kg/m^2^)	27.09 ± 5.89	28.74 ± 5.01	0.141	27.23 ± 5.77	29.31 ± 4.75	0.081
WC (cm)	90.29 ± 14.99	96.15 ± 15.46	0.092	90.72 ± 15.42	98.41 ± 14.05	**0.032**
**Blood pressure**						
SBP (mmHg)	121.04 ± 11.39	127.57 ± 12.32	**0.017**	121.89 ± 12.08	129.09 ± 10.98	**0.014**
DBP (mmHg)	75.83 ± 7.88	78.57 ± 8.00	0.130	76.15 ± 7.27	79.32 ± 9.54	0.213
**Metabolic profile**						
Fasting Glucose (mg/dL)	104.38 ± 15.61	116.57 ± 15.23	**0.001**	106.26 ± 16.32	118.54 ± 13.64	**0.001**
Total cholesterol (mg/dL)	177.54 ± 28.14	218.54 ± 44.67	**<0.001**	186.74 ± 37.16	217.27 ± 44.26	**0.009**
HDL cholesterol (mg/dL)	46.97 ± 11.77	40.94 ± 16.83	**0.007**	47.00 ± 13.94	37.31 ± 13.27	**0.002**
LDL cholesterol (mg/dL)	106.02 ± 27.74	147.13 ± 44.21	**<0.001**	114.99 ± 35.51	146.55 ± 46.31	**0.013**
Triglycerides (mg/dL)	122.73 ± 37.34	152.37 ± 55.33	**0.050**	123.75 ± 41.61	167.05 ± 50.36	**0.003**
**Bioelectrical variables**						
R (Ω)	502.08 ± 75.14	530.89 ± 84.95	0.125	511.97 ± 79.39	520.50 ± 84.01	0.693
Xc (Ω)	45.89 ± 7.56	37.40 ± 9.78	**<0.001**	43.70 ± 8.09	38.45 ± 12.02	**0.008**
PhA (°)	5.26 ± 0.62	4.00 ± 0.65	**<0.001**	4.93 ± 0.80	4.16 ± 0.86	**<0.001**
**Nutritional assessment**						
PREDIMED score	7.56 ± 1.28	4.29 ± 1.87	**<0.001**	6.69 ± 2.05	4.77 ± 2.22	**<0.001**
Total energy (kcal)	2223.29 ± 235.67	2307.88 ± 235.47	0.107	2256.96 ± 231.39	2264.49 ± 260.66	0.943
**Protein (g of total kcal)**	94.59 ± 12.21	101.77 ± 16.08	**0.026**	96.21 ± 12.29	101.55 ± 18.66	0.191
Animal (g of total kcal)	70.31 ± 11.54	73.77 ± 10.25	0.129	71.68 ± 11.11	71.99 ± 11.26	0.903
Plant (g of total kcal)	24.29 ± 10.01	28.01 ± 14.32	0.236	24.52 ± 10.42	29.54 ± 15.51	0.178
**Carbohydrate (g of total kcal)**	300.19 ± 36.57	308.89 ± 34.06	0.246	304.80 ± 36.50	301.25 ± 33.58	0.706
Complex (g of total kcal)	188.41 ± 23.05	190.63 ± 22.83	0.633	190.53 ± 23.27	186.06 ± 21.80	0.431
Simple (g of total kcal)	111.78 ± 15.77	118.27 ± 14.01	**0.040**	114.27 ± 15.53	115.19 ± 15.02	0.785
**Fat (gr of total kcal)**	71.57 ± 7.60	73.91 ± 9.57	0.275	72.55 ± 7.66	72.59 ± 10.73	0.879
SFA (g of total kcal)	25.79 ± 5.16	26.80 ± 10.30	0.664	26.03 ± 6.55	26.72 ± 10.46	0.829
MUFA (g of total kcal)	30.94 ± 3.65	31.34 ± 3.50	0.587	31.12 ± 3.36	31.07 ± 4.19	0.877
PUFA (g of total kcal)	14.84 ± 3.94	15.77 ± 5.80	0.566	15.39 ± 5.34	14.79 ± 2.90	0.791
n-6 PUFA (g/day)	7.64 ± 3.58	8.83 ± 4.86	0.173	8.21 ± 4.65	7.96 ± 2.57	0.500
n-3 PUFA (g/day)	7.20 ± 1.59	6.94 ± 1.98	0.415	7.18 ± 1.67	6.83 ± 1.99	0.299

A *p* value in bold type denotes a significant difference (*p* < 0.05). Abbreviation: GEP-NET, Gastroenteropancreatic Neuroendocrine Tumor; G, grading; BMI, Body Mass Index; WC, Waist Circumference; SBP, Systolic Blood Pressure; DBP, Diastolic Blood Pressure; HDL, High-Density Lipoprotein; LDL, Low-Density Lipoprotein; R, Resistance; Xc, Reactance; PhA, Phase angle; PREDIMED, PREvención con DIeta MEDiterránea; SFA, Saturated Fatty Acids; MUFA, MonoUnsaturated Fatty Acids; PUFA, PolyUnsaturated Fatty Acids.

**Table 4:** Demographic, clinical, metabolic and bioelectrical parameters in the GEP-NET patients according to disease status.

Parameters	Disease Status			
	Free of the Disease *n*. 34	Stable Disease *n*. 28	Progressive Disease *n*. 21	*p*-Value
**Age (years)**	55.61 ± 17.33	57.71 ± 15.23	56.95 ± 13.51	0.720
**Anthropometric measurement**				
BMI (kg/m^2^)	27.21 ± 4.94	27.54 ± 6.72	29.06 ± 4.83	0.411
WC (cm)	90.93 ± 16.20	92.36 ± 14.44	96.26 ± 15.36	0.458
**Blood pressure**				
SBP (mmHg)	123.08 ± 12.55	121.79 ± 11.07	127.62 ± 12.16	0.256
DBP (mmHg)	76.02 ± 7.66	77.14 ± 7.75	78.33 ± 8.99	0.631
**Metabolic profile**				
Fasting Glucose (mg/dL)	105.38 ± 12.99	106.82 ± 16.87	119.81 ± 17.42	**0.005**
Total cholesterol (mg/dL)	195.67 ± 41.66	178.64 ± 32.72	215.05 ± 42.99	**0.017**
HDL cholesterol (mg/dL)	42.15 ± 13.94	49.18 ± 12.43	41.81 ± 14.35	**0.035**
LDL cholesterol (mg/dL)	126.23 ± 38.67	105.35 ± 33.54	142.70 ± 44.65	**0.012**
Triglycerides (mg/dL)	136.50 ± 41.33	120.57 ± 38.41	152.71 ± 62.74	0.327
**Bioelectrical variables**				
R (Ω)	512.71 ± 83.84	507.61 ± 73.76	525.52 ± 85.06	0.767
Xc (Ω)	41.65 ± 9.88	45.79 ± 7.76	38.76 ± 9.84	**0.025**
PhA (°)	4.66 ± 0.82	5.19 ± 0.71	4.22 ± 0.91	**<0.001**
**Nutritional assessment**				
PREDIMED score	6.91 ± 2.25	6.54 ± 1.79	4.52 ± 2.02	**<0.001**
Total energy (kcal)	2256.94 ± 257.14	2254.24 ± 204.66	2268.51 ± 257.36	0.983
**Protein (g of total kcal)**	96.82 ± 13.50	97.39 ± 14.13	99.22 ± 16.36	0.855
Animal (g of total kcal)	71.87 ± 10.06	71.94 ± 10.98	71.37 ± 13.18	0.948
Plant (g of total kcal)	24.96 ± 10.62	25.44 ± 10.98	27.85 ± 15.63	0.752
**Carbohydrate (g of total kcal)**	302.62 ± 37.01	303.83 ± 30.79	305.91 ± 40.54	0.954
Complex (g of total kcal)	188.00 ± 23.11	189.85 ± 19.44	190.84 ± 27.26	0.902
Simple (g of total kcal)	114.61 ± 15.60	113.98 ± 14.46	115.07 ± 16.62	0.979
**Fat (g of total kcal)**	73.24 ± 9.49	72.15 ± 6.69	72.00 ± 9.31	0.868
SFA (g of total kcal)	27.26 ± 7.87	25.67 ± 5.12	25.24 ± 10.17	0.380
MUFA (g of total kcal)	30.88 ± 3.76	31.40 ± 3.22	31.10 ± 3.86	0.824
PUFA (g of total kcal)	15.10 ± 6.12	15.08 ± 2.96	15.65 ± 4.53	0.653
n-6 PUFA (gr/day)	7.99 ± 5.08	7.71 ± 2.77	8.97 ± 4.21	0.385
n-3 PUFA (gr/day)	7.11 ± 1.99	7.38 ± 1.03	6.68 ± 2.10	0.280

A *p* value in bold type denotes a significant difference (*p* < 0.05). Abbreviation: GEP-NET, Gastroenteropancreatic Neuroendocrine Tumor; BMI, Body Mass Index; WC, Waist Circumference; SBP, Systolic Blood Pressure; DBP, Diastolic Blood Pressure; HDL, High-Density Lipoprotein; LDL, Low-Density Lipoprotein; R, Resistance; Xc, Reactance; PhA, Phase angle; PREDIMED, PREvención con DIeta MEDiterránea; SFA, Saturated Fatty Acids; MUFA, MonoUnsaturated Fatty Acids; PUFA, PolyUnsaturated Fatty Acids.

**Table 5:** Bivariate proportional odds ratio model performed to assess the association of tumor aggressiveness with demographic, clinical, metabolic, and bioelectrical parameters.

Parameters	Grading G2				Stage IV (Metastases)			
	OR	*p*-Value	95% CI	R^2^	OR	*p*-Value	95% CI	R^2^
**Age (years)**	1.02	0.217	0.99–1.05	0.019	1.02	0.359	0.98–1.05	0.010
**Anthropometric measurement**								
BMI (kg/m^2^)	1.06	0.191	0.97–1.15	0.021	1.07	0.145	0.98–1.17	0.026
WC (cm)	0.07	**0.049**	0.99–1.06	0.036	1.03	**0.049**	1.00– 1.07	0.048
**Blood pressure**								
SBP (mmHg)	1.05	**0.018**	1.01–1.09	0.071	1.05	**0.020**	1.00–1.10	0.069
DBP (mmHg)	1.04	0.125	0.99–1.11	0.029	1.05	0.114	0.99–1.12	0.031
**Metabolic profile**								
Fasting Glucose (mg/dL)	1.06	**0.002**	1.02–1.09	0.136	1.05	**0.005**	1.01–1.09	0.108
Total cholesterol (mg/dL)	1.03	**<0.001**	1.02–1.05	0.241	1.02	**0.004**	1.00–1.04	0.108
HDL cholesterol (mg/dL)	0.97	**0.044**	0.94–1.00	0.045	0.93	**0.011**	0.88–0.98	0.105
LDL cholesterol (mg/dL)	1.03	**<0.001**	1.02–1.05	0.242	1.02	**0.003**	1.00–1.04	0.114
Triglycerides (mg/dL)	1.01	**0.007**	1.00–1.03	0.094	1.02	**0.001**	1.00–1.04	0.157
**Bioelectical variables**								
R (Ω)	1.00	0.109	0.99–1.01	0.032	1.00	0.667	0.99–1.00	0.002
Xc (Ω)	0.88	**<0.001**	0.83–0.95	0.200	0.94	**0.031**	0.89–0.99	0.060
PhA (°)	0.01	**<0.001**	0.01–0.08	0.529	0.31	**0.001**	0.15–0.64	0.149
**Nutritional assessment**								
PREDIMED score	0.31	**<0.001**	0.19–0.50	0.486	0.66	**0.001**	0.51–0.85	0.137
Total energy (kcal)	1.00	0.113	1.00–1.01	0.031	1.00	0.898	0.99–1.00	0.000
**Protein (g of total kcal)**	1.01	**0.029**	1.00–1.07	0.061	1.02	**0.042**	0.99–1.06	0.026
Animal (g of total kcal)	1.03	0.164	0.98–1.07	0.024	1.00	0.909	0.96–1.05	0.000
Plant (g of total kcal)	1.03	0.171	0.99–1.07	0.023	1.03	**0.002**	0.99–1.08	0.033
**Carbohydrate (g of total kcal)**	1.00	0.272	0.99–1.02	0.015	0.99	0.686	0.98–1.01	0.002
Complex (g of total kcal)	1.00	0.661	0.98–1.02	0.002	0.99	0.430	0.97–1.01	0.008
Simple (g of total kcal)	1.03	**0.042**	0.99–1.06	0.044	1.00	0.808	0.97–1.04	0.001
**Fat (gr of total kcal)**	1.03	0.218	0.98–1.09	0.019	1.00	0.983	0.95–1.06	0.000
SFA (g of total kcal)	1.02	0.555	0.96–1.08	0.004	1.01	0.136	0.95–1.08	0.002
MUFA (g of total kcal)	1.03	0.610	0.91–1.17	0.003	0.99	0.954	0.87–1.14	0.000
PUFA (g of total kcal)	1.04	0.391	0.95–1.14	0.009	0.97	0.614	0.87–1.09	0.003
n-6 PUFA (g/day)	1.07	0.081	0.96–1.20	0.020	0.99	0.807	0.87–1.11	0.001
n-3 PUFA (g/day)	0.92	0.502	0.71–1.18	0.005	0.89	0.428	0.68–1.18	0.008

A *p* value in bold type denotes a significant difference (*p* < 0.05). Abbreviation: BMI, Body Mass Index; WC, Waist Circumference; SBP, Systolic Blood Pressure; DBP, Diastolic Blood Pressure; HDL, High-Density Lipoprotein; LDL, Low-Density Lipoprotein; R, Resistance; Xc, Reactance; PhA, Phase angle; PREDIMED, PREvención con DIeta MEDiterránea; SFA, Saturated Fatty Acids; MUFA, MonoUnsaturated Fatty Acids; PUFA, PolyUnsaturated Fatty Acids; OR, Odds Ratio; IC, Interval Confidence.

**Table 6:** Multinomial logistic regression model to assess the association between disease status with age, anthropometric measurement, blood pressure, metabolic profile, bioelectrical variables and nutritional assessment.

	Progressive Disease		
Parameters	χ^2^	*p* Value	AIC
Age (years)	107.35	0.054	201.30
**Anthropometric measurement**			
BMI (kg/m^2^)	176.49	0.125	317.39
WC (cm)	156.71	**0.033**	266.41
**Blood pressure**			
SBD (mmHg)	28.64	**0.050**	75.82
DBD (mmHg)	23.06	**0.027**	56.61
**Metabolic profile**			
Fasting Glucose (mg/dL)	116.57	**0.043**	215.48
Total cholesterol (mg/dL)	140.08	0.220	277.92
HDL cholesterol (mg/dL)	109.25	**0.011**	187.09
LDL cholesterol (mg/dL)	168.17	0.147	309.55
Triglycerides (mg/dL)	159.85	**0.030**	269.70
**Bioelectical variables**			
R (Ω)	156.03	0.233	303.33
Xc (Ω)	82.00	0.088	173.49
PhA (°)	85.71	**0.010**	154.83
**Nutritional assessment**			
PREDIMED score	32.24	**0.005**	70.80
Total energy (kcal)	173.72	0.217	326.77
Protein (g of total kcal)	179.26	0.196	332.00
Animal (g of total kcal)	179.23	0.195	332.01
Plant (g of total kcal)	179.25	0.197	331.09
Carbohydrate (g of total kcal)	178.25	0.196	332.08
Complex (g of total kcal)	179.26	0.199	332.00
Simple (g of total kcal)	176.26	0.191	331.00
Fat (g of total kcal)	173.72	0.217	326.77
SFA (g of total kcal)	176.48	0.206	329.38
MUFA (g of total kcal)	173.72	0.217	326.77
PUFA (g of total kcal)	176.49	0.176	325.39
n-6 PUFA (gr/day)	176.50	0.206	329.39
n-3 PUFA (gr/day)	176.48	0.085	309.38

A *p* value in bold type denotes a significant difference (*p* < 0.05). Abbreviation: BMI, Body Mass Index; WC, Waist Circumference; SBP, Systolic Blood Pressure; DBP, Diastolic Blood Pressure; HDL, High-Density Lipoprotein; LDL, Low-Density Lipoprotein; R, Resistance; Xc, Reactance; PhA, Phase angle; PREDIMED, PREvención con DIeta MEDiterránea; SFA, Saturated Fatty Acids; MUFA, MonoUnsaturated Fatty Acids; PUFA, PolyUnsaturated Fatty Acids.

**Table 7:** Multiple regression analysis models (stepwise method) with tumor aggressiveness and nutritional parameters.

Parameters	Multiple Regression Analysis			
**Model 1–Tumor Grading-**	**R^2^**	**β**	**t**	***p* value**
PREDIMED score	0.591	−0.724	−9.45	**<0.001**
PhA (°)	0.595	−0.394	−4.04	**<0.001**
Simple carbohydrate (g of total kcal)	0.629	0.194	2.88	**0.005**
LDL cholesterol (mg/dL)	0.646	0.171	2.19	**0.031**
Variable excluded: WC, SBP, fasting Glucose, total cholesterol, HDL cholesterol, triglycerides, Xc, protein (g of total kcal).				
**Model 2–Tumor Stage-**	**R^2^**	**β**	**t**	***p* value**
Triglycerides (mg/dL)	0.151	0.402	3.95	**<0.001**
PREDIMED score	0.232	−0.306	−3.08	**0.003**
Variable excluded: WC, SBP, DBP, fasting Glucose, HDL cholesterol, PhA.				
**Model 3–Disease Status-**	**R^2^**	**β**	**t**	***p* value**
PREDIMED score	0.152	−0.403	−3.96	**<0.001**
Variable excluded: WC, SBP, DBP, fasting Glucose, HDL cholesterol, triglycerides, PhA.				

A *p* value in bold type denotes a significant difference (*p* < 0.05). Abbreviation: PREDIMED, PREvención con DIeta MEDiterránea; PhA, Phase angle; LDL, Low-Density Lipoprotein; WC, Waist Circumference; SBP, Systolic Blood Pressure; HDL, High-Density Lipoprotein; Xc, Reactance; DBP, Diastolic Blood Pressure.

## Supplementary Materials

The following are available online at http://www.mdpi.com/2072-6643/10/12/1854/s1, Table S1: Total energy and daily macronutrients/micronutrients intake of GEP-NET patients and control group, Table S2: Grading, disease stage and disease status in GEP-NET patients according to gender, gender, smoking, physical activity and PREDIMED categories, Table S3: Correlations of ki67% index and tumor size with demographic, clinical, metabolic and bioelectrical parameters, Table S4: Multiple regression analysis models (stepwise method) with the tumor aggressiveness and nutritional parameters.
