# Supplementary material for: Impact of Nutritional Status on Gastroenteropancreatic Neuroendocrine Tumors (GEP-NET) Aggressiveness
Source: Nutrients. 2018 Dec 1;10(12):1854. doi: 10.3390/nu10121854 (PMC6316835; doi:10.3390/nu10121854)
Supplement: Supplementary file 1 [file nutrients-10-01854-s001.pdf]

Table S1. Total energy and daily macronutrients/micronutrients intake of GEP-NET patients and control group.

Parameters	NET patients n = 83	Control group n = 83	p-value
Total energy (kcal)	2258.96 ± 237.88	2296.31 ± 280.05	0.399
Protein (gr of total kcal)	97.62 ± 14.33	101.82 ± 16.34	0.102
Animal (gr of total kcal)	71.77 ± 11.08	70.86 ± 12.51	0.498
Plant (gr of total kcal)	25.86 ± 12.09	30.96 ± 11.78	**0.003**
Carbohydrate (gr of total kcal)	303.86 ± 35.58	309.20 ± 38.62	0.381
Complex (gr of total kcal)	189.34 ± 22.84	206.81 ± 27.74	**<0.001**
Simple (gr of total kcal)	114.52 ± 15.31	102.39 ± 13.38	**<0.001**
Fat (gr of total kcal)	72.56 ± 8.51	72.48 ± 10.13	0.832
SFA (gr of total kcal)	26.21 ± 7.71	23.29 ± 3.46	0.060
MUFA (gr of total kcal)	31.11 ± 3.57	33.03 ± 4.91	**0.009**
PUFA (gr of total kcal)	15.23 ± 4.80	15.35 ± 2.90	0.384
n-6 PUFA (gr/day)	8.14 ± 4.18	5.24 ± 2.89	**<0.001**
n-3 PUFA (gr/day)	7.09 ± 1.76	10.11 ± 1.91	**<0.001**

In spite of no differences in energy intake between the two groups, GEP-NET patients consumed a lower quantity of plant protein, complex carbohydrate, MUFA and n-3 PUFA, and higher quantity of simple carbohydrate and n-6 PUFA than control individuals. A p value in bold type denotes a significant difference ($p < 0.05$). Abbreviation: GEP-NET, Gastroenteropancreatic Neuroendocrine Tumor; SFA, Saturated Fatty Acids; MUFA, MonoUnsaturated Fatty Acids; PUFA, PolyUnsaturated Fatty Acids.

Table S2. Grading, disease stage and disease status in GEP-NET patients according to gender, gender, smoking, physical activity and PREDIMED categories.

Parameters	Grading		Disease Stage		Disease status		
	**G1 n.48**	**G2 n.35**	**Stage I-III n.61**	**Stage IV n.22**	**Free of the disease n. 34**	**Stable disease n. 28**	**Progressive disease n. 21**
**Gender**							
Males	21, 52.5	19, 47.5	26, 65.0	14, 35.0	14, 35.0	14, 35.0	14, 35.0
Females	27, 62.8	16, 37.2	35, 81.4	8, 18.6	20, 46.5	14, 32.6	9, 20.9
	χ^2=0.53, *p*=0.468		χ^2=2.08, *p*=0.149			χ^2=2.13, *p*=0.344	
**Smoking**							
Yes	18, 56.3	14, 43.8	21, 65.6	11, 34.4	17, 53.1	5, 15.6	10, 31.3
No	30, 58.8	21, 41.2	40, 78.4	11, 21.6	17, 33.3	23, 45.1	11, 21.6
	χ^2=0.00, *p*=0.997		χ^2=1.06, *p*=0.303			χ^2=1.67, *p*=0.322	
**Physical activity**							
Yes	27, 65.9	14, 34.1	33, 80.5	8, 19.5	18, 43.9	14, 34.1	9, 22.0
No	21, 50.0	21, 50.0	28, 66.7	14, 33.3	16, 38.1	14, 33.3	12, 28.6
	χ^2=1.54, *p*=0.215		χ^2=1.39, *p*=0.239			χ^2=0.54, *p*=0.766	
**PREDIMED categories**							
Low adherence to the MD	2, 6.7	28, 93.3	17, 56.7	13, 43.3	8, 26.7	7, 23.3	15, 50.0
Average adherence to the MD	42, 85.7	7, 14.3	40, 81.6	9, 18.4	23, 46.9	20, 40.8	6, 12.2
High adherence to the MD	4, 100	0, 0	4, 100	0, 0	3, 75.0	1, 25.0	0, 0
	**χ^2=50.74, *p*<0.001**		**χ^2=7.47, *p*=0.024**			**χ^2=16.38, *p*=0.003**	

When classified GEP-NET patients based on tumor grade G1/G2, stage and disease status, we observed that the majority of patients with aggressive disease (GEP-NET G2, stage IV and progressive disease) presented a low adherence to the MD according to PREDIMED categories. No significant differences were observed when classified these patients for gender, smoking and physical activity. A *p* value in bold type denotes a significant difference ($p < 0.05$). Abbreviation: GEP-NET, Gastroenteropancreatic Neuroendocrine Tumor; PREDIMED, PREvención con DIetaMEDiterránea; **MD,** Mediterranean Diet.

Table S3. Correlations of ki67% index and tumor size with demographic, clinical, metabolic and bioelectrical parameters.

**Parameters**	**ki67% index**				**Tumor size (mm)**			
	**Simple correlation**		**After adjusting for BMI and WC**		**Simple correlation**		**After adjusting for BMI and WC**	
	r	***p*-value**	*r*	***p*-value**	*r*	***p*-value**	*r*	***p*-value**
**Age (years)**	0.212	0.055	0.116	0.301	0.157	0.175	0.104	0.377
**Anthropometric measurement**								
BMI (kg/m^2)	0.338	**0.002**	-	-	0.178	0.124	-	-
WC (cm)	0.394	**<0.001**	-	-	0.197	0.089	-	-
**Blood pressure**								
SBP (mmHg)	0.325	**0.003**	0.044	0.699	0.236	**0.040**	0.198	0.091
DBP (mmHg)	0.183	0.098	-0.043	0.700	0.044	0.705	-0.085	0.474
**Metabolic profile**								
Fasting Glucose (mg/dL)	0.456	**<0.001**	0.365	**0.001**	0.198	0.087	0.125	0.291
Total cholesterol (mg/dL)	0.522	**<0.001**	0.492	**<0.001**	0.168	0.147	0.130	0.268
HDL cholesterol (mg/dL)	-0.386	**<0.001**	-0.313	**0.004**	-0.271	**0.018**	-0.239	**0.040**
LDL cholesterol (mg/dL)	0.499	**<0.001**	0.470	**<0.001**	0.190	0.101	0.159	0.177
Triglycerides (mg/dL)	0.370	**<0.001**	0.258	**0.020**	0.145	0.213	0.090	0.447
**Bioelectrical variables**								
R (Ω)	0.036	0.745	0.036	0.753	0.030	0.798	0.024	0.839
Xc (Ω)	-0.676	**<0.001**	-0.666	**<0.001**	-0.288	**0.012**	-0.254	**0.029**
PhA (°)	-0.867	**<0.001**	-0.864	**<0.001**	-0.359	**0.001**	-0.320	**0.005**
**Nutritional assessment**								
PREDIMED score	-0.861	**<0.001**	-0.849	**<0.001**	-0.430	**<0.001**	-0.396	**<0.001**
Total energy (kcal)	0.115	0.302	0.138	0.220	0.102	0.379	0.116	0.323
**Protein (gr of total kcal)**	0.152	0.170	0.143	0.201	0.241	**0.036**	0.246	**0.035**
Animal (gr of total kcal)	0.112	0.312	0.178	0.112	0.149	0.197	0.188	0.109
Plant (gr of total kcal)	0.115	0.303	0.035	0.757	0.071	0.542	0.054	0.648
**Carbohydrate (gr of total kcal)**	0.069	0.534	0.081	0.470	0.078	0.502	0.126	0.283
Complex (gr of total kcal)	0.008	0.942	-0.010	0.928	0.096	0.409	0.094	0.428
Simple (gr of total kcal)	0.150	0.177	0.207	0.063	0.127	0.275	0.156	0.183
**Fat (gr of total kcal)**	0.108	0.332	0.166	0.137	-0.079	0.495	-0.059	0.619
SFA (gr of total kcal)	0.007	0.947	0.062	0.583	-0.181	0.118	-0.159	0.175
MUFA (gr of total kcal)	0.077	0.486	0.076	0.499	-0.007	0.954	-0.010	0.931
PUFA (gr of total kcal)	0.050	0.653	0.046	0.684	0.079	0.498	0.077	0.514
n-6 PUFA (gr/day)	0.121	0.275	0.104	0.353	-0.038	0.743	0.046	0.697
n-3 PUFA (gr/day)	-0.055	0.619	-0.081	0.471	0.061	0.600	-0.044	0.708

Ki-67% showed significant correlations with all anthropometric measurements and metabolic profile, SBP, PhA and PREDIMED score. After adjusting for BMI and WC, all correlations were maintained, except for SBP. Tumor size significant correlated with SBP, HDL cholesterol, PhA, PREDIMED score, and protein consumption. After adjusting for BMI and WC, all correlations were maintained, except for SBP. A *p* value in bold type denotes a significant difference ($p < 0.05$). Abbreviation: BMI, Body Mass Index; WC, Waist Circumference; SBP, Systolic Blood Pressure; DBP, Diastolic Blood Pressure; HDL, High-Density Lipoprotein; LDL, Low-Density Lipoprotein; R, Resistance; Xc, Reactance; PhA, Phase angle; PREDIMED, PREvención con DIeta MEDiterránea; SFA, Saturated Fatty Acids; MUFA, MonoUnsaturated Fatty Acids; PUFA, PolyUnsaturated Fatty Acids.

Table S4. Multiple regression analysis models (stepwise method) with the tumor aggressiveness and nutritional parameters.

**Parameters**	**Multiple Regression analysis**			
Model 1 –ki67%-	**R^2**	***β***	**t**	***p* value**
PhA (°)	0.306	−0.560	−6.09	**<0.001**
Variable excluded: fasting Glucose, total cholesterol, HDL cholesterol, LDL cholesterol, triglycerides, Xc, PREDIMED score				
Model 2 –Tumor size-	R^2	***β***	**t**	***p* value**
PREDIMED score	0.153	−0.405	−3.81	**<0.001**
Variable excluded: HDL cholesterol, Xc, PhA, protein (gr of total kcal)				

Among the parameters correlated with ki67% (model 1), and tumor size (model 2), ki67% and tumor size were well predicted by PhA and PREDIMED score, respectively. A *p* value in bold type denotes a significant difference ($p < 0.05$). Abbreviation: PhA, Phase angle; HDL, High-Density Lipoprotein; LDL, Low-Density Lipoprotein; Xc, Reactance; PREDIMED, PREvención con DIeta MEDiterránea.
